# Exploring the therapeutic potential of *Hibiscus rosa sinensis* synthesized cobalt oxide (Co_3_O_4_-NPs) and magnesium oxide nanoparticles (MgO-NPs)

**DOI:** 10.1016/j.sjbs.2021.05.035

**Published:** 2021-05-21

**Authors:** Kainat, Muhammad Aslam Khan, Farhad Ali, Shah Faisal, Muhammad Rizwan, Zahid Hussain, Nasib Zaman, Zobia Afsheen, Muhammad Nazir Uddin, Nadia Bibi

**Affiliations:** a Institute of Biotechnology and Microbiology, Bacha Khan University, Charsadda, KPK, Pakistan; b Department of Biological Sciences, International Islamic University, Islamabad, Pakistan; c Center for biotechnology and microbiology university of swat, KPK, Pakistan; d Department of Microbiology and Biotechnology, Abasyn University, Peshawar, KPK, Pakistan; e Department of Microbiology, Shaheed Benazir Bhutto Women University, Peshawar, KPK, Pakistan

**Keywords:** *Hibiscus rosa sinensis*, Cobalt oxide nanoparticles, Magnesium oxide nanparticles, Urinary tract infections, Green synthesis

## Abstract

Herein, we present a green, economic and ecofriendly protocol for synthesis of cobalt oxide (Co_3_O_4_-NPs) and magnesium oxide nanoparticles (MgO-NPs) for multifaceted biomedical applications. In the study, a simple aqueous leaf extract of *Hibiscus rosa sinensis,* was employed for the facile one pot synthesis of Co_3_O_4_-NPs and MgO-NPs. The well characterized NPs were explored for multiple biomedical applications including bactericidal activity against urinary tract infection (UTI) isolates, leishmaniasis, larvicidal, antidiabetic antioxidant and biocompatibility studies. Our results showed that both the NPs were highly active against multidrug resistant UTI isolates as compared to traditional antibiotics and induced significant zone of inhibition against *Proteus Vulgaris, Pseudomonas Aurigenosa and E.coli.* The NPs, in particular Co_3_O_4_-NPs also showed significant larvicidal activity against the *Aedes Aegypti,* the mosquitoes involve in the transmission of Dengue fever. Similarly, excellent leishmanicidal activity was also observed against both the promastigote and amastigote forms of the parasite. Furthermore, the particles also exhibited considerable antidiabetic activity by inhibiting α-amylase and α-glucosidase enzymes. The biosynthesized NPs were found to be excellent antioxidant and biocompatible nanomaterials. Owing to ecofriendly synthesis, non-toxic and biocompatible nature, the *Hibiscus rosa sinensis* synthesized Co_3_O_4_-NPs and MgO-NPs can be exploited as potential candidates for multiple biomedical applications.

## Graphical abstract

**Figure:**
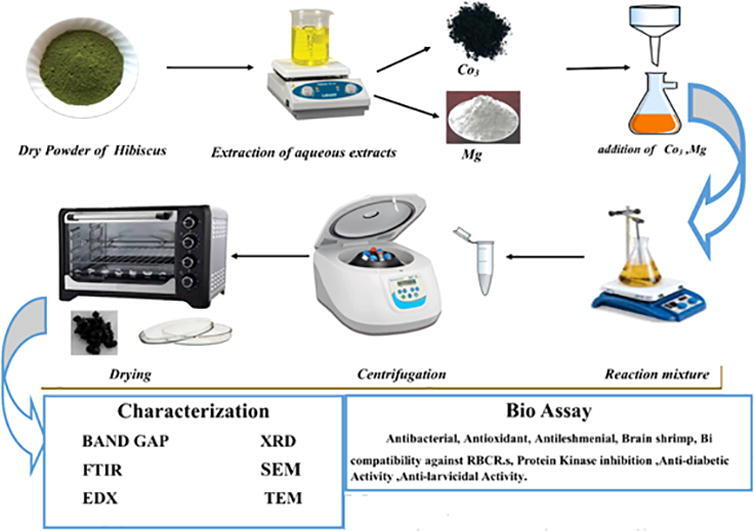

## 1 Introduction

Metallic nanoparticles (MNPs) have fascinated scientific community since decades and are being heavily exploited in nanotechnology. Unique plasmonic and physicochemical characteristics such as high stability, reactivity and excellent photothermal properties make metallic nanoparticles ideal materials to be exploited in wide spectrum applications in energy, environment and medicine (Singh et al. 2020).

Since the last decade, cobalt oxide nanoparticles (Co_3_O_4_-NPs) and magnesium oxide nanoparticles (MgO-NPs) have gained tremendous attention because of their unique characteristics and diverse applications in materials sciences, physics and chemistry (Ansari, 2017, Ansari, 2017, Behzadi et al., 2019). Both the metallic nanoparticles are widely used in catalysis, nanosensors, biodiesel synthesis, waste water treatment and optoelectronic devices and in biomedical applications (Manjari et al., 2017, Manjari, 2017, Verma and Kumar, 2019).

Recently, both the MNPs have also been exploited for various therapeutic applications such as antimicrobial, anticancer, biosensor, cellular labeling and as carrier for drugs and biomolecules and as enhancement agents in magnetic resonance imaging (Aisida et al., 2019, Aisida et al., 2020a, Aisida et al., 2020b).

Traditionally, Co_3_O_4_-NPs and MgO-NPs are prepared using physical and chemical methods. Physical methods include laser ablation, electrolysis, evaporation- condensation diffusion, sputter deposition, high energy ball milling and pyrolysis. While the chemicals methods include multiple approaches such as chemical reduction, electrochemical and thermal decomposition. However, there are numerous drawbacks associated with such approaches like high energy consumption, high costs, waste of chemicals and most importantly may prove hazardous both in lab and to environment. Contrary to physical and chemical methods, green synthesis is considered as safe, inexpensive, rapid, ecofriendly and easy to scale up (Madubuonu et al., 2020, Muhammad et al., 2019, Yaqoob et al., 2020, Jan et al., 2020a).

Owing to advantages provided by plants as biological source for synthesis of nanoparticles, we have used *Hibiscus rosa sinensis.* The herb which belong to the *Malvaceae* family, is widely cultivated in the tropical regions mainly as an ornamental plant. Traditionally, the plant has numerous medicinal uses for treating fever and coughs, diabetes, healing of wounds, bacterial and fungal infections and gastric ulcers (Braglia et al., 2010, Melzer et al., 2013, Missoum, 2018, Muhammad et al., 2019).

In the current study, a simple aqueous leaf extract of *Hibiscus rosa sinensis* was used to synthesize Co_3_O_4_-NPs and MgO-NPs*.* Though several types of MNPs such as Au-NPs, Ag-NPs, ZnO-NPs and ZnCr_2_O_4_/ZnCrO have been synthesized from aqueous extract of *Hibiscus rosa sinensis* yet no or limited biomedical applications have been reported (Philip, 2010, Devi and Gayathri, 2014, Mayedwa et al., 2021) As per thorough literature review, it is the first ever report on the herb for the synthesis of Co_3_O_4_-NPs and MgO-NPs for diverse biomedical applications. In the study, we have comparatively analyzed both the NPs, coated with/without antibiotics for Urinary tract infection (UTI) isolates. Moreover, the literature lacks enough data regarding the larvicidal potential of the green synthesized Co_3_O_4_-NPs and MgO-NPs. Keeping in view such research gap, the well characterized NPs were investigated for multifaceted biomedical applications.

## 2 Material and methods

### 2.1 Extract preparation from *Hibiscus rosa sinensis*

The native herb was collected from District Charsadda and was taxonomically verified at Department of Botany, Bacha Khan University, Khyber Pakhtunkhwa, Pakistan. Briefly, the shade dried leaves were grounded into fine powder in a grinding machine. 30 g of the powder was then added into 300 mL distilled water in a 500 mL flask and was boiled for 20 min at 100 °C. The mixture was then incubated overnight at 37 °C in a shaker (150 rpm) for maximum extraction. The mixture was then filtered twice using Whatman filter paper grade 1 and the extract was stored at 4 °C for further processing.

### 2.2 Biosynthesis of cobalt oxide nanoparticles (Co_3_O_4_-NPs)

Cobalt Oxide nanoparticles (Co_3_O_4_*-*NPs) were biosynthesized using a previously reported protocol with some alterations (Khalil et al. 2020). In brief, 3 g of Cobalt Acetate tetrahydrate (C_4_H_6_CoO_4_; Sigma Aldrich) was added to 50 mL *Hibiscus Rosa sinensis* aqueous extract (pH: 5.6) in 250 mL flask. The mixture was stirred at 60 °C for 2 h on magnetic stirrer with hot plate. To collect the nanoparticles, the mixture was allowed to cool down after reaction, following centrifugation at 10,000 rpm for 10 min. With subsequent centrifugation, the supernatant was discarded while the pellet collected was further washed thrice with distilled water. The final, well washed pellet was then casted into petri plate, oven dried at 80 °C, grinded into fine powder in pastor and mortar and finally calcinated at 400 °C for 2 h.

### 2.3 Biosynthesis of magnesium oxide nanoparticles (MgO-NPs)

Magnesium Oxide nanoparticles (MgO*-*NPs) were biosynthesized using similar method as used for the synthesis of Co_3_O_4_-NPs with minor change. In brief, 3 g of Magnesium sulfate heptahydrate (MgSO_4_·7H_2_O; Sigma Aldrich) was added to 50 mL *Hibiscus Rosa sinensis* aqeous extract (pH: 5.6) in a 250 mL flask. Like, Co_3_O_4_-NPs, the mixture was stirred for 2 h at 60 °C on magnetic stirrer with hot plate. To collect the nanoparticles, the mixture was allowed to cool down after reaction, following centrifugation at 10,000 rpm for 10 min. With subsequent centrifugation, the supernatant was discarded while the pellet collected was further washed thrice with distilled water. The final, well washed pellet was then casted into petri plate followed by oven drying at 80 °C. The dried material was grounded into fine powder in pastor and mortar and calcinated at 400 °C for 2 h in furnance.

### 2.4 Physicochemical and morphological characterization

The fine calcinated powder was utilized for multiple characterization techniques including UV–Vis, X-Ray Diffraction (XRD), Fourier Transform Infrared (FTIR) spectroscopy, Scanning Electron Microscopy (SEM), Transmission electron microscope (TEM), Energy Dispersive X-Ray Analysis (EDX) and thermal gravimetric analysis (TGA). The biosynthetic reaction was monitored using UV–Visible in the range 200 to 700 nm while the chemical nature and surface chemistry of the synthesized particles was evaluated using FTIR in the range from 400 cm^−1^ to 4000 cm^−1^. Similarly, the phase structure of the particles was investigated by analyzing XRD patterns (Aisida et al., 2020).

Morphology and size of the particles were investigated using SEM and TEM at a voltage 200 kV. A thin film of each synthesized Co_3_O_4_-NPs and MgO-NPs was prepared by dropping a small amount on the carbon grid and were visualized under the microscope. Similarly, elemental composition was evaluated using Energy Dispersive X-Ray Spectroscopy (DX) attached to the SEM. While thermal properties of the NPs were studied using thermal gravimetric analysis in the range of 25 °C-600 °C.

### 2.5 Biological applications

#### 2.5.1 Antibacterial activity of Co_3_O_4_-NPs and MgO-NPs

The antibacterial activity of Co_3_O_4_-NPs and MgO-NPs in pristine as well as coated with antibiotics was investigated against multi drug resistant strains of urinary tract isolates (UTI). A disc diffusion method was used against *Klebsiella pneumonae*, *Echerichia coli*, *Pseudomonas aeruginosa* and *Staphylococcus aureus* which were isolated from UTI patients, collected at Hayat Abad medical Complex Peshawar (HMC), Pakistan.

#### 2.5.2 Co_3_O_4_-NPs and MgO-NPs coated antibiotic discs preparation

The antibiotics coated Co_3_O_4_-NPs and MgO-NPs discs were made by suspending 20 mg of the NPs separately in 1 mL distilled water in an eppendorf tube. Aliquot of 5 μL was taken from the stock solution and poured onto antibiotic disc within petri plates, followed by oven drying at 80 °C for 20 min. The same procedure was applied to each tested antibiotic disc.

#### 2.5.3 Disc diffusion assay

The standard Kirby Bauer disc diffusion method was employed to examine the bactericidal potential of both coated and non-coated antibiotics against the tested MDR urinary tract isolates. According to the standard, seeding density (1 × 106 CFU/mL) of the tested isolates were adjusted. From the refreshed stock cultures, 50 μL were poured onto the solidified nutrient agar plates, to prepare the bacterial lawn. Following, coated and non– coated antibiotic discs were carefully placed in the petri plates and the plates were placed for incubation for 24 h at 37 °C. After the incubation period, the plates were observed for antibacterial potential of the samples by measuring the zone of inhibition induced by the samples in mm using vernier caliper.

### 2.6 Anti-larvicidal activity

Anti-larvicidal potential of Co_3_O_4_-NPs and MgO-NPs was investigated against the third instar larvae of dengue vector i.e. *Aedes aegypti* L. The well standard protocol previously used was employed with minor alterations (Salem et al. 2020). Briefly, five groups of which four groups (for different concentrations) each containing 25 third instar larvae, and one a control group were used. The assay was carried out in a sterilized well plate with each well containing 25 third instar larvae in either 200 mL of the test solution at the desired concentration or simple distilled water as negative control. The well plate was incubated at standard insectary conditions i.e. for 12 h light to 12 h dark photoperiod, under 28 °C temperature and relative humidity of 80%. During the experiment, the larvae were not feed. After 24 h percent mortality was examined. Those specimens were counted as dead when, which were not able to move or moved sluggishly by stimulation of touch. The assay was carried out in five replicates, and the % mortality represents the mean of five triplicates.

### 2.7 Anti-leishmanial assay

The anti-lesihmanial activity of the Co_3_O_4_-NPs and MgO-NPs was investigated against the parasite Leishmania tropica (KWH23) in both promastigote and amastigote forms. In brief, both the parasitic forms were freshly cultured in MI99 medium supplemented with 10% FBS for overnight incubation period. In a 96 well plate, 20 µL of tested sample and 180 µL aliquot from the refreshed culture were gently mixed followed by an incubation period of 72 h at 25 °C. Dimethyl sulfoxide (DMSO) and Amphotericin-B and DMSO, prepared in PBS (1%) were used as positive and negative controls, respectively. Following the incubation period, 20 µL MTT solution (2 mg/0.5 mL in dH_2_0) was added to an each tested well and the plate was re-incubated for another 4 h at room temperature (25 °C ± 2). Using a microplate reader absorbance of the tested samples were taken 540 nm via and % inhibition was calculated using the formula as:

$$\%Inhibition=\left[1-\left\{\frac{Abs}{Abc}\right\}\right]\times 100$$

Abs and Abc refers to the absorbance of the tested sample and negative control used in the experiment. Different concentration of the tested samples were used in the bioassay and potential IC_50_ values were calculated using Table curve 2D v5. 01.

### 2.8 Antioxidant assays

#### 2.8.1 Total antioxidant capacity determination (TAC)

The assay was carried out using well optimized previously reported methodology with couple of alterations (Ihsan et al. 2021). In the experiment, in an eppendorf tube an aliquot of 100 µL of the sample was mixed with 900 mL of TAC reagent (0.6 M sulphuric acid, 28 mM sodium phosphate and 4 mM ammonium molybdate, in 50 mL dH_2_0). The mixture was incubated in water bath for 2 h at 80 °C, allowed to cool followed by absorption recording at 630 nm. The assay was carried out thrice and TAC activity was expressed as µg ascorbic acid equivalent (AAE) per mg of the test sample.

#### 2.8.2 Total reducing power (TRP)

The assay was performed using a methodology followed in the literature (Aziz et al. 2020b). In brief, a reaction mixture was prepared in an eppendorf tube containing 100 µL of the test sample, 400 µL phosphate buffer (pH 6.6) and 100 µL of potassium ferric cyanide (1% w/v), followed by water bath incubation at 55 °C for 30 min. After the incubation period, 400 µL of trichloroacetic acid (10% w/v) was gently mixed to the mixture, centrifuged at 4000 rpm for 12 min. 140 µL of the supernatant was carefully taken, poured into corresponding well in the plate, that already contained, 60 µL of ferric cyanide solution (0.1% w/v). Using a microplate reader absorbance of the samples were measured at 630 nm and the reducing power (TRP) was calculated as AAE (ascorbic acid equivalent) per mg the tested sample.

#### 2.8.3 Free radical scavenging assay (FRSA)

The possible DPPH free radical scavenging ability was determined using a standard bioassay in multiple concentrations ranging from 12.5 µL to 400 µL (Jan et al., 2020). Briefly, 10 µL of the test sample was mixed with 190 µL of the DPPH reagent in each well of a 96 well plate followed by dark incubation at 37° C for 1 h. After the incubation period, absorbance were recorded 525 nm, and the free radical scavenging potential of each sample was calculated using the formula

$$\left(\%\right)FRSA=\left(1-\frac{\mathrm{Absample}}{\mathrm{Absnegativecontrol}}\right)\times 100$$

#### 2.8.4 ABTS assay

Trolox antioxidant assay or ABTS activity using a previously described method (Tagliazucchi et al. 2010) was also carried out. In brief, an ABTS reaction solution was prepared by mixing ABTS salt (7 mM) and potassium per sulphate (2.45 mM) in 1:1. After through mixing, the mixture was incubated in dark for 15 h. After mixing with the test samples, the mixture was replaced in dark for 20 min at 27 °C. Using microplate reader (BioTek ELX800), absorbance of the samples was recorded at 734 nm. Dimethyl sulfoxide and Trolox were investigated as positive and negative controls, respectively and the antioxidant potential was depicted as TEAC.

### 2.9 Antidiabetic assay

#### 2.9.1 α-amylase inhibition assay

To investigate the α-amylase inhibition potential of Co_3_O_4_-NPs and MgO-NPs a protocol reported (Zohra et al., 2019, Ullah et al., 2021), was employed with few alterations. Briefly, in a 96 well plate 15 µL of the test sample, 25 µL of α-amylase and starch (40 µL) were mixed gently, followed by 40 min incubation at 50 °C. After the incubation period, 20 µL of IM HCL and 90 µL of iodine solution were added into each well, followed by absorbance measurement at 540 nm. In the assay, Acarbose served as positive control and DMSO as negative control. The inhibition was calculated as % using the formula

$$\%\alpha -amylaseinhibition=\left(\frac{Abs-Abnc}{Absb-Absnc}\right)\times 100$$

Where abs, abs b and Abs nc depicts absorbance of the sample, absorbance of the blank and absorbance of negative control.

#### 2.9.2 α-glucosidase inhibition assay

To augment the antidiabetic results, α-glucosidase inhibition assay was also performed using a well standard protocol (Jan et al., 2020). Briefly, a reaction mixture containing 10 μL of the test sample, 490 μL of PB (pH 6.8) and 250 μL of 5 mM ρ-nitrophenyl α-D-glucopyranoside was incubated for a brief time of 5 min at at 37 °C followed by addition of 250 μL α-glucosidase, and reincubation of 15 min at 37 °C. The reaction was then terminated by pouring 2 mL Na_2_CO_3_ (200 mM) solution, followed by absorptions recorded at 400 nm. The same controls as used in were used α-amylase inhibition were also utilized in the assay and the activity was calculated in percentage as follows

$$\%\alpha -amylaseinhibition=\left(\frac{Abs-Abnc}{Absb-Absnc}\right)\times 100$$

Where abs, absb and Absnc depicts absorbance of the sample, absorbance of the blank and absorbance of negative control.

### 2.10 Biocompatibility studies

Biocompatibility of Co_3_O_4_-NPs and MgO-NPs was evaluated against isolated human red blood cells (hRBCs), employing previously used methodology (Khalil et al. 2017). In the experiment, 2 mL of healthy blood was collected in EDTA tube, with an informed consent, from a volunteer. To isolate the erythrocytes, the collected blood was centrifuged at 13000 rpm for 10 min. After centrifugation, the supernatant was discarded and the pellet was washed thrice with PBS. After thorough washing, the pellet was mixed with 9.8 mL of PBS (pH: 7.2), with gentle shacking to prepare the PBS-erythrocyte suspension. Tested samples with various concentration and erythrocyte suspension were incubated for an hour in Eppendorf tubes at 35 °C, followed at 12,000 rpm for 8 min. From the centrifuged eppendorf tubes, 200 μL of the supernatant was transferred into corresponding well in a 96-well plate. Using a microplate reader, absorptions were measured for haemoglobin release at 540 nm. Triton X-100 (0.5) and Dimethyl Sulfoxide were employed as positive and negative controls. % hemolysis was calculated as

$$\left(\%\right)Haemolysis=\left(\frac{\mathrm{Abs}-Abnc}{\mathrm{Abpc}-Abnc}\right)\times 100$$

Where Abs, Abnc and Abpc denotes absorbance of sample, negative control and positive control respectively.

## 3 Results

### 3.1 Optical properties

**Fig. 1:**
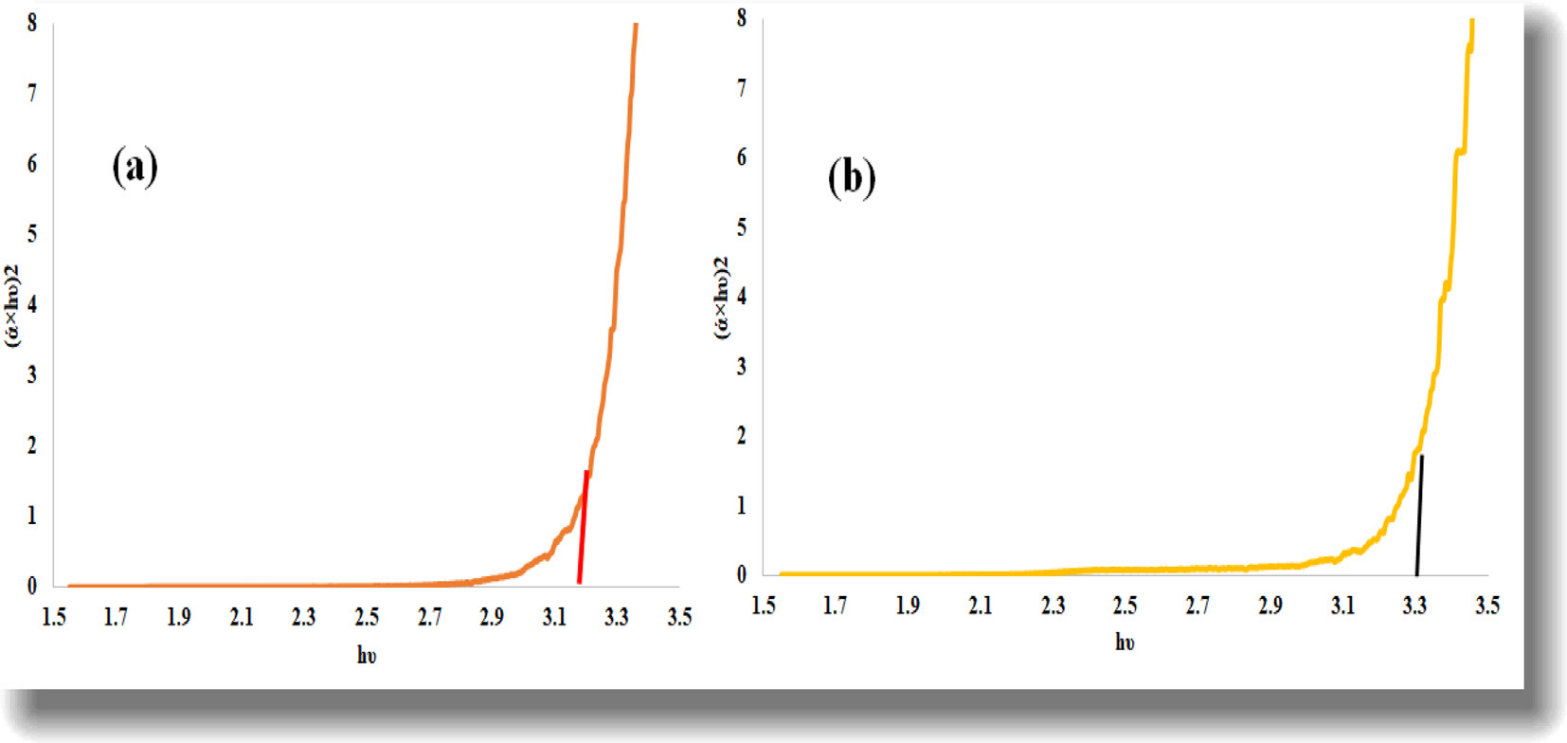
Band gap of *Hibiscus rosa sinensis* Co_2_O_3_-NPs and MgO-NPs.

The optical band gap of both Co_3_O_4_ and MgO nanoparticles were determined using the basic relationship between absorbance and incident photon energy (hν) given by Eq. (1) as shown in the Fig. 1. The band gap of Co_3_O_4_- NPs were calculated as 3.18 eV while that of MgO-NPs was determined as 3.3 eV respectively (Iqbal et al. 2020).

### 3.2 XRD analysis

**Fig. 2:**
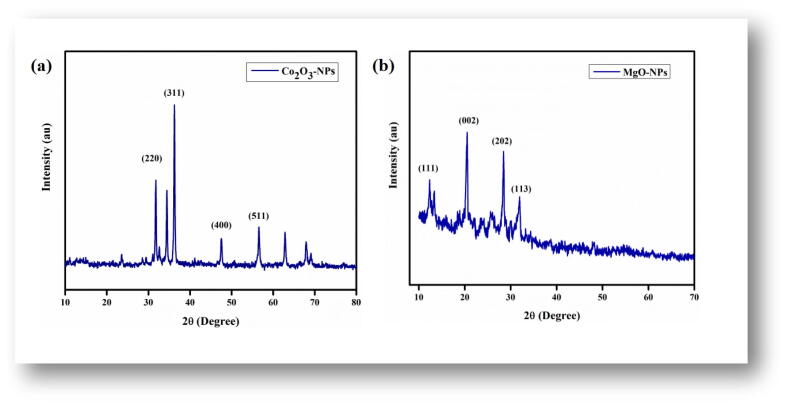
Typical XRD patterns of *Hibiscus rosa sinensis* (a) Co_3_O_4_-NPs, (b) MgO-NPs.

The XRD patterns were examined to verify the successful synthesis of both Co_3_O_4_-NPs and MgO -NPs as shown in Fig. 2a and 2b. The Co_3_O_4_-NPs resulted in multiple peaks, at 2θ i.e. 21.14, 26.84, 29.32, and 36.22, corresponding to the plane metallic cobalt indices of (1 1 1), (2 2 0), (3 1 1), and (2 2 2). Similar XRD patterns were also recorded in previous reports (Dutta et al., 2020, Shahzadi et al., 2019). The average crystallite size as determined using Debye Scherer equation was calculated as 29.6 nm respectively. While MgO-NPs exhibited clear peaks at 2θ 12.57°, 21.02°, 29.98°, 32.36, corresponding to miller’s indices (1 1 1), (0 0 2), (2 0 2) and (1 1 3), thus conforms the polycrystalline cubic structure (JCPDS No. 87–0653). No other impurities were detected in XRD patterns. The mean crystallite size is calculated using (0 0 2) reflection and found to be 31.6 nm.

### 3.3 FTIR analysis

**Fig. 3:**
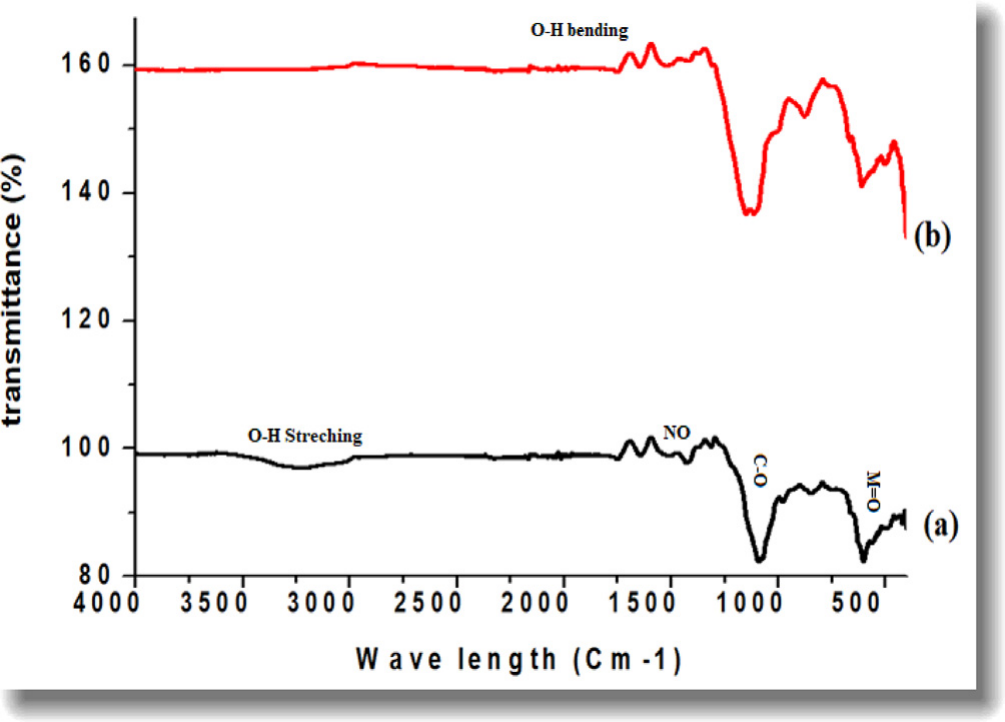
Typical FTIR spectra of *Hibiscus rosa sinensis* Co_3_O_4_-NPs and MgO-NPs.

FTIR was used to investigate the surface chemistry of the biosynthesized NPs as shown in the Fig. 3a and 3b. Numerous absorption peaks were recorded on varied wavelengths for Co_3_O_4_-NPs including at 3228 cm^−1^, 1747 cm^−1^, 1644 cm^−1^, 1423 cm^−1^, 1088 cm^−1^, 842 cm^−1^ and 598 cm^−1^. Absorption peak at 3228 cm^−1^, is corresponded to the O–H stretching vibrations, in hydroxyl groups (Priyadarshini et al. 2012). A peak at 1644 cm^−1^ indicates the C = O stretching vibrations, an indication of carbonyl group present that is present in carboxylic acids, ketones and esters (Kasthuri, xxxx, Kasthuri, 2009). A sharp peak at 1423 cm^−1^ is inferred–C = C– stretching vibrations in aromatic ring. Similarly, an absorption peak at 1088 cm^−1^ represents C–O–C stretching vibrations. A band at 598 cm^−1^ corresponds to the stretching vibrations of Co-O bond in Co_3_O_4,_ thus affirming the successful synthesis of Co_3_O_4_-NPs (Ren et al. 2009). On the other hand FTIR spectra of MgO displayed prominent peaks at 3473 cm^−1^, 1748 cm^−1^, 1642 cm^−1^, 1523 cm^−1^, 1148 cm^−1^, 876 cm^−1^ and 607 cm^−1^. Like in Co_3_O_4_-NPs, the absorption peaks at 3473 cm^−1^, 1748 cm^−1^, 1642 cm^−1^ are attributed to O-H stretching vibration in hydroxyl groups, C = O stretching vibrations and C–O–C stretching vibrations, respectively (Pugazhendhi, 2019). The peaks at 1523 cm^−1^ and 1148 cm-1 corresponded to O-H bending and C-O stretching of the functional groups in primary alcohols. While the broad absorption peak on 607 cm^−1^ indicated Mg-O bond stretching affirming the successful synthesis of MgO-NPs (Umaralikhan and Jaffar, 2018). The FTIR spectra, thus confirms the successful capping of plant metabolites on the surface of Co_3_O_4_-NPs and MgO-NPs.

### 3.4 Morphological studies and elemental analysis

**Fig. 4:**
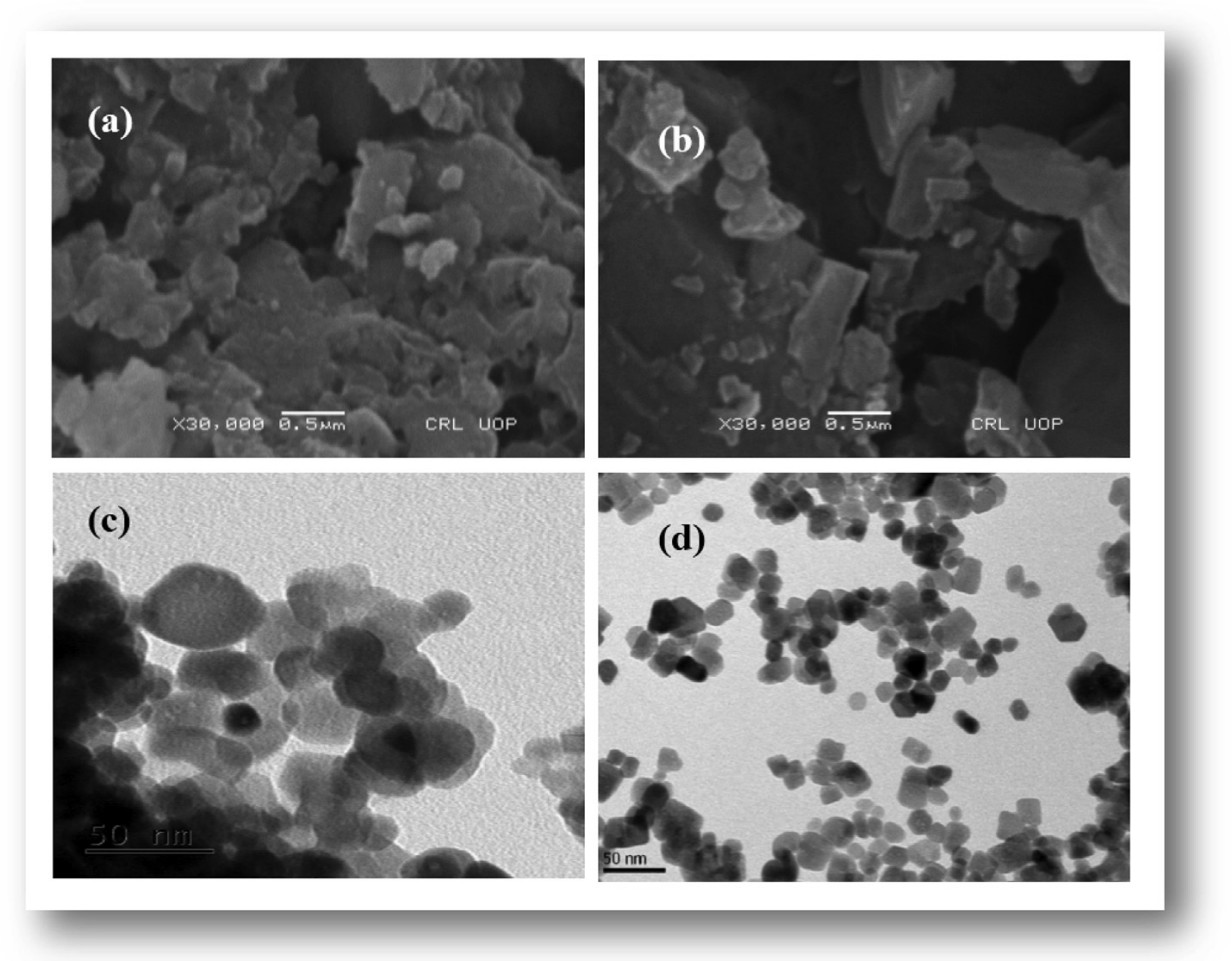
Typical SEM and TEM micrograph of (a and c) for Co_3_O_4_-NPs, (b and d) for MgO-NPs.

**Fig. 5:**
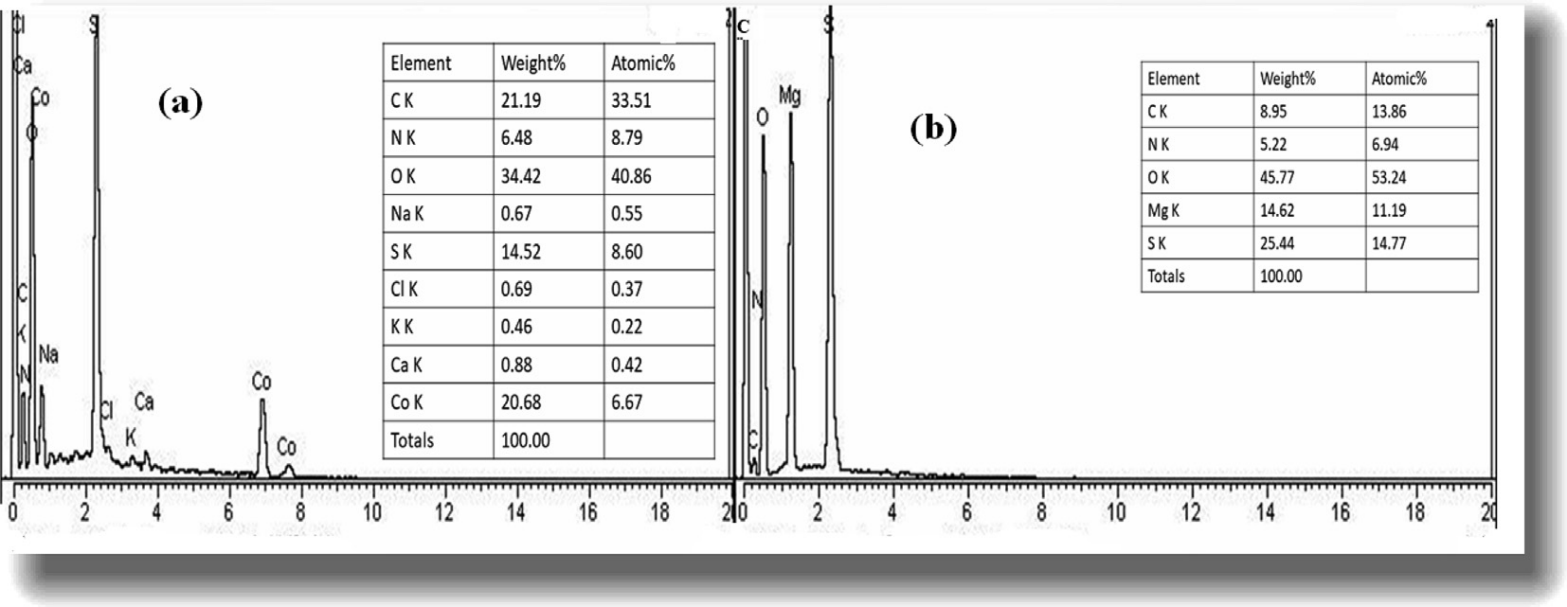
Typical EDX spectrum of (a) Co_3_O_4_-NPs (b) MgO-NPs.

Morphology and elemental analysis of the biosynthesized NPs were examined using SEM, TEM and EDX. As shown in the Fig. 4. SEM micrographs of both the Co_3_O_4_-NPs and MgO-NPs revealed agglomerated morphology with larger mean size of the powdered samples. Using well sonicated samples, TEM micrographs showed spherical or elliptical morphology of both the synthesized NPs with mean size of 27.72 ± 10.32 for MgO-NPs and 18.98 ± 8.45 for Co_3_O_4_ NPs. The dimensions of about 50 particles were calculated for each sample, using ImageJ software. Similar morphologies were also observed in previous literature (Koyyati et al., 2016, Sushma et al., 2016). The EDX data (Fig. 5) revealed that both the metal oxide NPs exhibited high percentage of Cobalt and Magnesium along with high percentage of oxygen as shown in the figure. The carbon atom seen in the spectrum is due to the residual organic biomolecules present in the sample from the plant extract which served as reducing and capping agents to produce the NPs.

### 3.5 Thermal properties

**Fig. 6:**
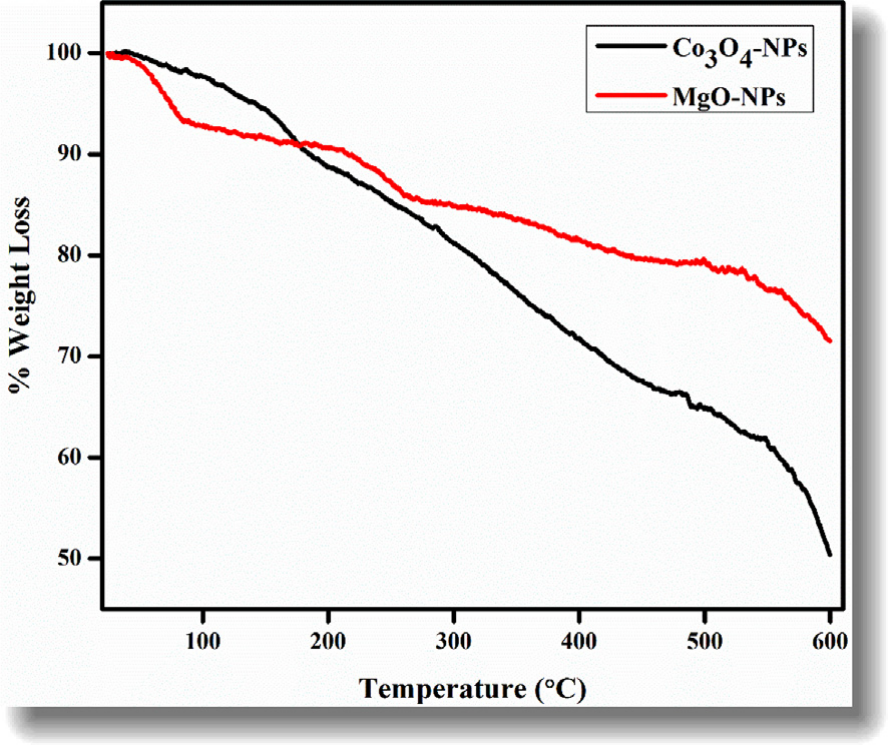
Typical TGA thermogram of *Hibiscus rosa sinensis* Co_3_O_4_-NPs and MgO-NPs.

Thermal properties of the biosynthesized nanoparticles were studied using thermal gravimetric (TGA) analysis in the temperature range from 25 °C up to 600 °C as shown in Fig. 6. The total weight loss of the Co_3_O_4_-NPs were recorded as 50% till 600 °C while MgO-NPs resulted in only 27% weight loss. The initial weight loss up to 150 °C is attributed to the dehydration and loss of moisture content from the samples (Jeevanandam et al. 2020). Our results thus indicated that biosynthesized MgO-NPs are thermally more stable as compared to Co_3_O_4_-NPs.

## 4 Biomedical applications

### 4.1 Antibacterial activity of Co_3_O_4_-NPs and MgO-NPs against MDR urinary tract isolates

**Fig. 7:**
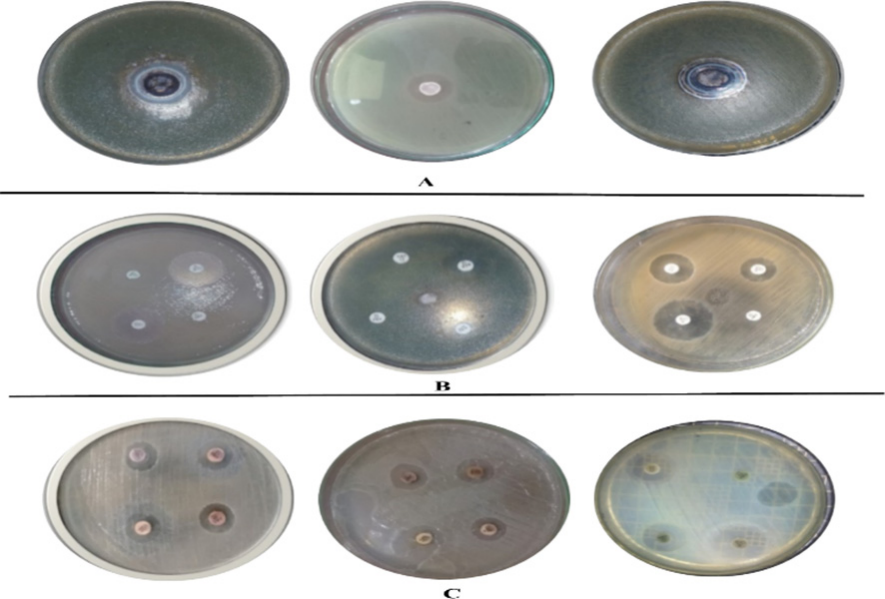
A) activity of **Co_3_O_4_-NPs** and **MgO-NPs** B) activity of antibiotics. C) activity of **Co_3_O_4_-NPs** and **MgO-NPs** coated with antibiotics.

Bactericidal activity of pristine Co_3_O_4_-NPs and MgO-NPs, pristine antibiotics as well as antibiotics coated Co_3_O_4_-NPs and MgO-NPs were investigated against the urinary tract infection (UTI) isolates, including *Proteus Vulgaris*, *Pseudomonas Aurigenosa and E.coli* (Table 1) and (Fig. 7). 1% Co_3_O_4_-NPs showed the highest activity against multi drug resistant (MDR) *Proteus Vulgaris* with 21 ± 1.32 mm zone of inhibition, followed by MgO-NPs that induced 22 ± 1.17 mm inhibition zone. Similarly, the pristine NPs also exhibited significant activity against *Pseudomonas Aurigenosa and E.coli* as shown in the table. Contrary to pristine NPs, all the strains shown significant resistant to tested pristine antibiotics i.e. Meropenem, Imipenem, oxacillin and Ciprofloxacin. However, it was observed that the activity of NPs coated antibiotics increased to some extent against *Pseudomonas Aurigenosa, E.Coli*. and *Proteus Vulgaris*. For instance, the inhibition activity of pristine MgO-NPs was recorded as 19 ± 1.38 mm against *E.coli* while that of pristine Ciprofloxacin was measured as 15 ± 1.2 mm. On the other hand, MgO-NPs coated Ciprofloxacin resulted in 23 ± 1.1 mm ZOI, a 38.1% increase in the inhibition activity. Our finding thus concludes that the biosynthesized Co_3_O_4_-NPs and MgO-NPs, not only exhibit significant bactericidal activity in pristine form but there activity can also be augmented against Multidrug Drug Resistant (MDR) strains by coating antibiotics.

**Table 1:** Antibacterial activity of Co_3_O_4_-NPs and MgO-NPs against UTI isolates.

Bacterial Strains	NPs	ZOI of NPs	Antibiotics	CLSI	ZOI of Non-coated Antibiotics	ZOI of Coated NPs	% increased potency of coated NPs
*Pseudomonas* *Aurigenosa*	**Co_3_O_4_-NPs**	20 ± 1.47	MEM	18	13 ± 0.8	17 ± 0.7	22.2
			IPM	22	11 ± 1.0	16 ± 0.6	22.7
			OX	17	08 ± 0.3	14 ± 1.0	35.4
			CIP	21	14 ± 0.7	20 ± 1.1	28.6
	**MgO-NPs**	19 ± 1.38	MEM	18	13 ± 1.0	19 ± 0.9	33.4
			IPM	22	11 ± 1.3	15 ± 0.6	18.2
			OX	17	08 ± 0.2	13 ± 0.4	29.5
			CIP	21	14 ± 0.5	19 ± 1.1	23.9
*Proteus Vulgaris*	**Co_3_O_4_-NPs**	21 ± 1.32	MEM	18	11 ± 0.9	19 ± 1.1	44.5
			IPM	22	13 ± 1.1	20 ± 1.4	31.9
			OX	17	10 ± 0.5	18 ± 1.6	47.1
			CIP	21	15 ± 0.8	22 ± 1.2	47.4
	**MgO-NPs**	22 ± 1.25	MEM	18	11 ± 0.4	20 ± 1.0	50.0
			IPM	22	13 ± 1.0	22 ± 0.9	41.0
			OX	17	10 ± 0.7	16 ± 1.0	35.3
			CIP	21	15 ± 0.6	23 ± 0.6	38.1
*E. coli*	**Co_3_O_4_-NPs**	16 ± 1.13	MEM	18	12 ± 0.9	17 ± 1.1	27.7
			IPM	22	14 ± 1.0	18 ± 0.23	18.2
			OX	17	10 ± 0.7	13 ± 0.9	17.7
			CIP	21	15 ± 0.4	20 ± 1.0	23.8
	**MgO-NPs**	19 ± 1.61	MEM	18	12 ± 0.6	18 ± 0.7	33.4
			IPM	22	14 ± 1.1	22 ± 1.3	36.4
			OX	17	10 ± 0.8	15 ± 0.6	29.4
			CIP	21	15 ± 1.2	23 ± 1.1	38.1

### 4.2 Larvicidal activity

**Fig. 8:**
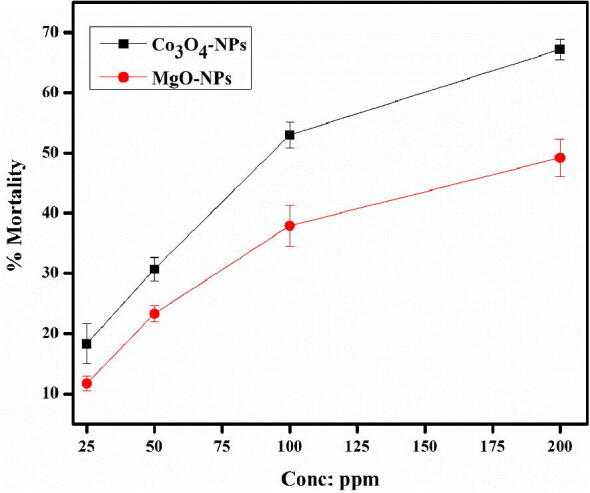
% larvicidal (Mortality) activity of *Hibiscus rosa sinensis* Co_3_O_4_-NPs and MgO-NPs.

In the present study, biosynthesized Co_3_O_4_-NPs and MgO-NPs were tested against third instar *A. aegypti* larvae to evaluate the potential of NPs as a tool to control the Dengue. The NPs were evaluated at varied concentrations ranging from 25 ppm to 200 ppm. In general both the NPs showed excellent but dose dependent larvicidal activity with Co_3_O_4_-NPs displaying relatively significant potential as shown in Fig. 8. At the highest concentration (200 ppm) Co_3_O_4_-NPs resulted in 67.2 ± 1.7% mortality of the larvae while lowest mortality of 18.3 ± 5.3% was observed at 25 ppm. On the other hand, MgO-NPs resulted in 49.2 ± 8.1% mortality at 400 ppm and 11.7 ± 1.3% mortality at the lowest tested concentration of 25 ppm.

### 4.3 Anti-leishmanial activity

**Fig. 9:**
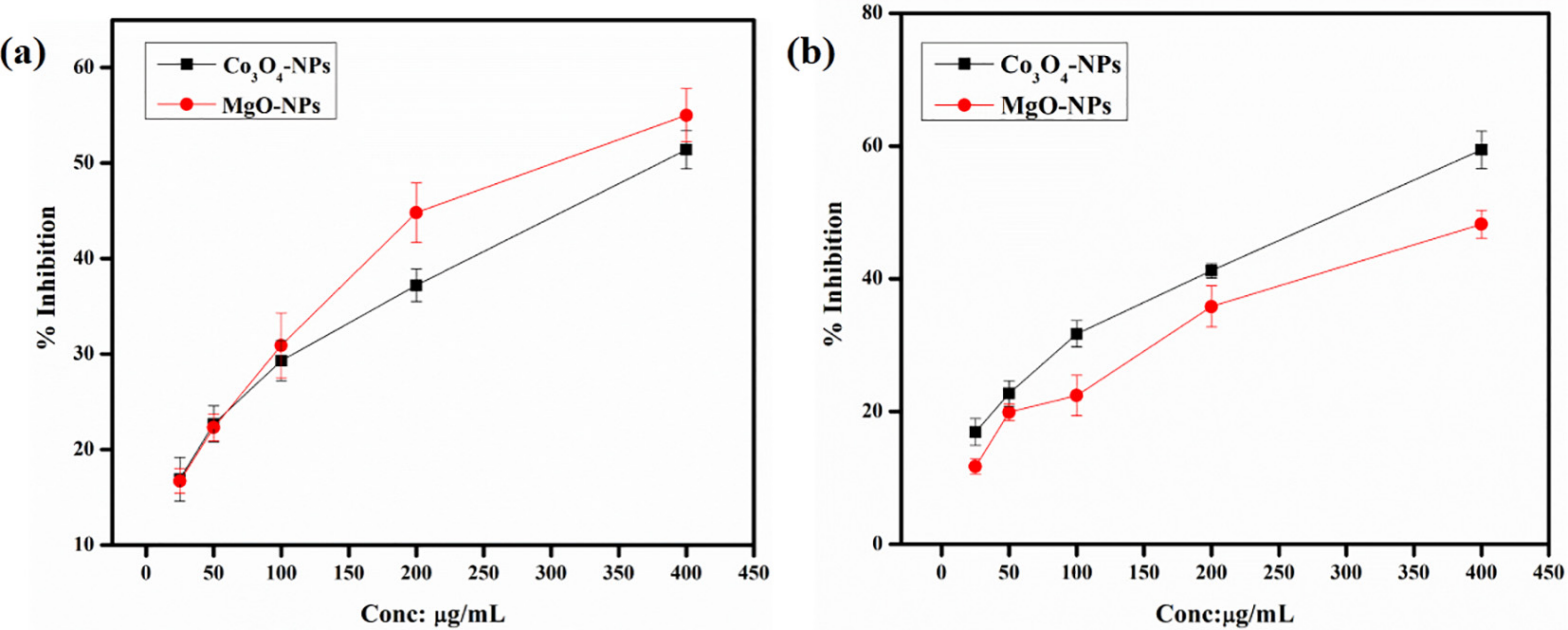
**%** Mortality activity of *Hibiscus rosa sinensis* Co_3_O_4_-NPs and MgO-NPs against amastigote and promastigote form of *Leishmania tropica.*

The biosynthesized Co_3_O_4_-NPs and MgO-NPs were tested against both the promastigote and amastigote cultures of the parasite i.e. *Leishmania tropica* using MTT bio assay. The results are illustrated in the Fig. 8 and 9. As can be seen in the figure, a dose dependent activity was observed for both the NPs. Co_3_O_4_-NPs displayed the highest activity against the promastigote with 57.32 ± 1.48% mortality at 400 µg/mL as compared to MgO-NPs which resulted in 44 ± 1.48% mortality. The amastigote also showed varied susceptibly to both the NPs, for instance at the highest concentration, Co_3_O_4_-NPs resulted in 48 ± 2.24% mortality while MgO induced 54 ± 1.88% mortality.

### 4.4 Anti-diabetic activity

**Fig. 10:**
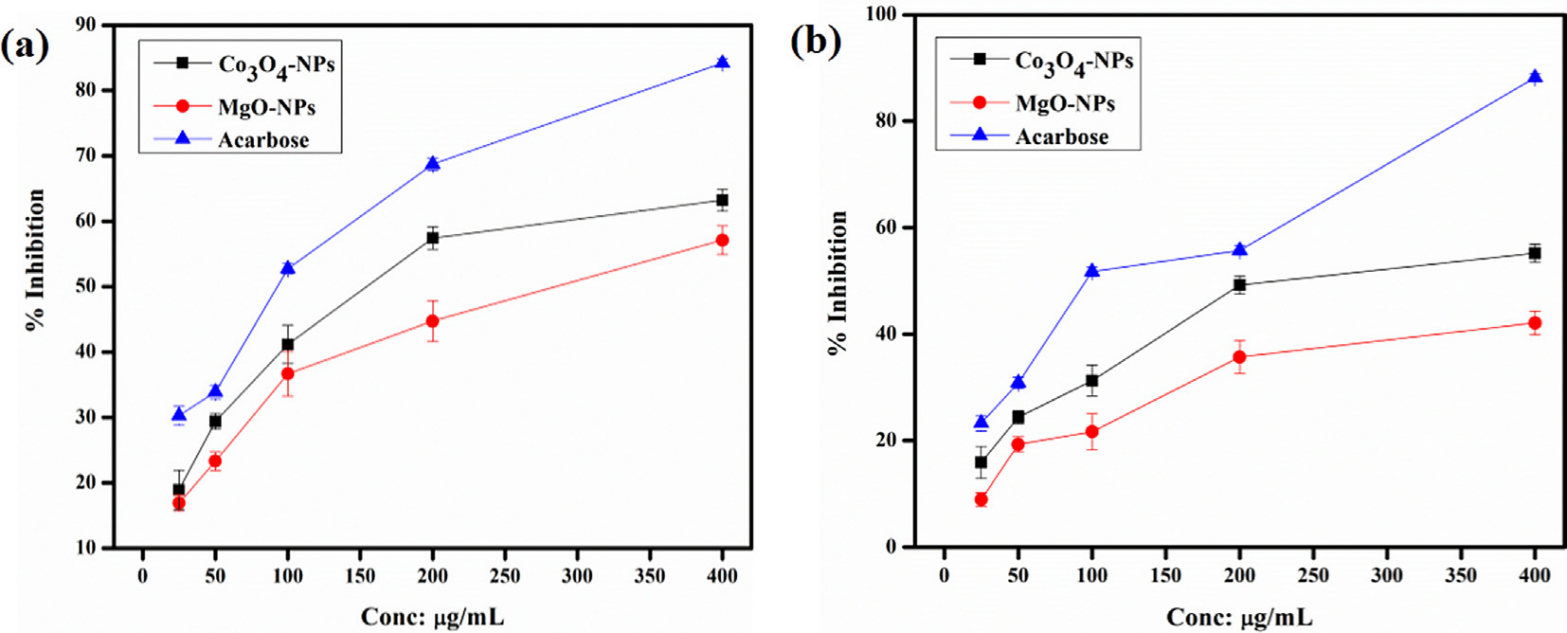
% Inhibition activity of *Hibiscus rosa sinensis* Co_3_O_4_-NPs and MgO-NPs against (a) α –amylase and (b) α –glucosidase.

In the study, both the NPs were subjected to α –amylase and α –glucosidase inhibitory potential, in varied concentrations. The results obtained are illustrated in Fig. 10. Our findings indicated, remarkable α-amylase and α-glucosidase inhibition potential of the NPs. At the highest tested concentration, Co_3_O_4_-NPs induced 63.19 ± 1.44% inhibition of α-amylase with IC_50_ of 298 ± 0.88 mg/mL while MgO-NPs resulted in 54.32 ± 2.0% inhibition with IC_50_ 327 ± 0.82 µg/mL. Similarly, Co_3_O_4_-NPs also exhibited significant inhibition potential against α-glucosidase as compared to MgO-NPs with 53.27 ± 0.84% inhibition (IC_50_: 357 ± 0.82 µg/mL). On the other hand, MgO-NPs induced 41.72 ± 0.24% inhibition with IC_50_: greater than 400 µg/mL.

### 4.5 Antioxidant assay

The *in vitro* antioxidant potential of Co_3_O_4_-NPs and MgO-NPs was screened using total antioxidant capacity (TAC), total reducing power (TRP), ABTS (2,2′-azino-bis(3-ethylbenzothiazoline-6-sulfonic acid) and DPPH (2, 2-diphenyl-1-picrylhydrazyl) free radical scavenging assays (FRSA). The results are illustrated in the Table 2, Table 3. In general, the prepared NPs displayed excellent antioxidant potential. Initially, phosphomolybdenum based bioassay was carried out test the total antioxidant capacity (TAC). The assay relies on reducing Mo (VI) to Mo (V) leading to a complex formation of phosphate-molybdate. The complex is identified by its green color and only form if the tested agent has the antioxidant potency (Aziz et al., 2020). Highest antioxidants capacity in terms of ascorbic acid equivalents was observed as for 78.91 ± 2.04 μg AAE/mg for Co_3_O_4_-NPs at 400 µg/mL while MgO-NPs showed comparatively less TAC activity of 68.92 ± 2.40 μg AAE/mg**.** TAC assay was supported with total reducing power estimation (TRP) technique. The assay relies on transformation of Fe^+3^ to Fe^+2^ ions. Like TAC activity, highest TRP was observed as 43.82 ± 2.2 μg for Co_3_O_4_-NPs. On the other hand, MgO-NPs displayed weak reducing potential of 37.93 ± 2.2 μg AAE/mg. Moreover, to augment the findings, ABTS and DPPH free radical scavenging assays were also employed. These spectrophotometric methods are based on appeasing the stabilized colored radicals of DPPH and ABTS demonstrating the scavenging potential of the tested material. In the study, excellent free radical scavenging activities of Co_3_O_4_-NPs and MgO-NPs was observed**.** Highest DPPH and ABTS free radical scavenging activity for Co_3_O_4_-NPs was calculated as 57 ± 0.42 (IC_50_; 330 µg/mL) and 78.30 ± 0.74 TEAC, respectively. On the other hand, MgO-NPs displayed relatively better radical scavenging potential with DPPH scavenging potential of 67.28 ± 0.39 (IC_50_; 320 µg/mL) and ABTS free radical scavenging activity of 53.20 ± 0.28 TEAC, respectively. From the results summarized, it was concluded that the biosynthesized NPs displayed excellent antioxidant potential in particular Co_3_O_4_-NPs as compared

**Table 2:** Antioxidant potential of *Hibiscus rosa sinensis* synthesized Co_3_O_4_-NPs.

Conc (µg/mL)	TAC (µg AAE/mg)	TRP (µg AAE/mg)	ABTC (CTEAC)	DPPH (% FRSA)
**400**	61.1 ± 0.83	43.41 ± 0.83	77.12 ± 0.28	56.3 ± 0.28
**200**	55.37 ± 0.27	39.51 ± 0.87	63.63 ± 0.39	41.1 ± 0.71
**100**	33.86 ± 0.72	22.23 ± 0.26	44.64 ± 0.56	27.69 ± 0.32
**50**	25.29 ± 0.76	16.76 ± 0.58	25.47 ± 0.26	18.45 ± 0.98
**25**	19.16 ± 0.25	07.41 ± 0.36	16.39 ± 0.15	10.19 ± 0.48
Conc (µg/mL)	TAC (µg AAE/mg)	TRP (µg AAE/mg)	ABTC (CTEAC)	DPPH (% FRSA)
**400**	61.1 ± 0.83	43.41 ± 0.83	77.12 ± 0.28	56.3 ± 0.28
**200**	55.37 ± 0.27	39.51 ± 0.87	63.63 ± 0.39	41.1 ± 0.71
**100**	33.86 ± 0.72	22.23 ± 0.26	44.64 ± 0.56	27.69 ± 0.32
**50**	25.29 ± 0.76	16.76 ± 0.58	25.47 ± 0.26	18.45 ± 0.98
**25**	19.16 ± 0.25	07.41 ± 0.36	16.39 ± 0.15	10.19 ± 0.48

**Table 3:** Antioxidant potential of *Hibiscus rosa sinensis* synthesized MgO-NPs.

Conc (µg/mL)	TAC (µg AAE/mg)	TRP (µg AAE/mg)	ABTC (TEAC)	DPPH (% FRSA)
400	49.39 ± 0.51	42 ± 0.62	53.14 ± 0.29	69.2 ± 0.23
200	31.37 ± 0.16	29.6 ± 0.20	49.63 ± 0.19	51.8 ± 0.82
100	25.47 ± 0.41	21.29 ± 0.71	38.64 ± 0.45	44.34 ± 0.48
50	19.29 ± 0.36	17.16 ± 0.62	24.47 ± 0.14	36.47 ± 0.82
25	10.21 ± 0.62	9.16 ± 0.42	18.39 ± 0.35	29.78 ± 0.59

### 4.6 Biocompatibility studies

To evaluate the potential toxicology, Co_3_O_4_-NPs and MgO-NPs were tested for hemolytic properties against human red blood cells (hRBCs). In the experiment, the test samples and freshly isolated human red blood cells (hRBCs) were incubated together in a buffer solution that mimics the *in vivo* cellular conditions. The assay relies on bursting of RBCs, leading to hemoglobin release in the media. The rupturing can be triggered only if the tested sample exhibit toxilogical potential to the cells. In convention, hemolysis of RBCs greater than 25% is considered hemolytic while hemolysis < 10% is referred to as non-hemolytic. Our studies, noted excellent hemocompatibility, even at the highest tested concentrations as shown in the Table 4. Both the samples, did not show any hemolytic activity on the tested concentrations while negligible hemolysis was observed for the NPs at 400 µg/mL.

**Table 4:** % hemolytic activity of *Hibiscus rosa sinensis* Co_3_O_4_-NPs and MgO-NPs.

Conc (µg/mL)	Co_3_O_4_-NPs % Hemolysis	MgO-NPs % Hemolysis
400	2.69 ± 0.47	2.53 ± 0.21
200	1.37 ± 0.25	1.21 ± 0.63
100	0.89 ± 0.23	0.67 ± 0.72
50	0.40 ± 0.51	0.35 ± 0.45

## 5 Discussion

In general, cobalt oxide (Co_3_O_4_-NPs) and magnesium oxide nanoparticles (MgO-NPs) are synthesized by multiple chemical and physical methods such as chemical vapor deposition, thermal evaporation, sol–gel, sonichemical and spray pyrolysis among others (Iravani and Varma, 2020, Abinaya et al., 2021). However, such procedures are high energy demanding, expensive, time consuming and are not eco-friendly (Vijayan et al. 2016). Moreover, chemical methods may result in adsorption of toxic chemicals on the surface of nanoparticles that may lead to adverse effects in biomedical applications (Jain et al. 2017). Biological methods that exploit living organism (microbes, plants) or living systems (enzymes) for the synthesis of nanoparticles is one possible alternative for ecofriendly and inexpensive synthesis of (Co_3_O_4_-NPs) and magnesium oxide nanoparticles (MgO-NPs).

In the current study, an aqueous extract of *Hibiscus rosa sinensis* was utilized as reducing and stabilizing agent for the synthesis of multifunctional (Co_3_O_4_-NPs) and magnesium oxide nanoparticles (MgO-NPs). To the best of our knowledge it is the first ever study on *Hibiscus rosa sinensis* Co_3_O_4_-NPs and MgO-NPs for diverse biomedical applications.

Previous studies have been indicated that *Hibiscus rosa sinensis* possess bioactive properties and is recommended to be used as an herbal alternative to cure multiple diseases including heart disorders, tumors, convulsion, diabetes, inflammation, oxidative stress, diarrhea and ulcers (Kandhare et al., 2012).

*Hibiscus rosa sinensis* synthesized Co_3_O_4_-NPs and MgO-NPs were characterized by UV–Vis spectroscopy, X-Ray Diffraction (XRD), Fourier Transform Infrared (FTIR) spectroscopy, Scanning Electron Microscopy (SEM), Transmission electron microscope (TEM), Energy Dispersive X-Ray Analysis (EDX) and thermal gravimetric analysis (TGA). After through, physicochemical and morphological characterization both the NPs were evaluated and compared for therapeutic properties, that included antibacterial potential against urinary tract infection (UTI) isolates, leishmaniasis, larvicidal, antidiabetic antioxidant and biocompatibility studies.

Urinary tract infections (UTIs) is amongst the most common bacterial infections that is affecting more than 150 million people globally, each year. Though UTIs affects both males and females, yet the infection occurs more commonly in women that could likely infect 50% during their lifespan. Current therapeutic approaches are suboptimal due to high prevalence of multidrug-resistant (MDR) uropathogens that make the antibiotic treatment for acute infection ineffective (McLellan and Hunstad 2016). In our study, both pristine Co_3_O_4_-NPs and MgO-NPs, showed excellent bactericidal activity against the urinary tract infection (UTI) isolates, including *Proteus Vulgaris*, *Pseudomonas Aurigenosa and E.coli.* Our finding thus concludes that the biosynthesized Co_3_O_4_-NPs and MgO-NPs, not only exhibit significant bactericidal activity in pristine form but there activity can also be augmented against Multidrug Drug Resistant (MDR) strains by coating antibiotics Our results are in harmony with previous studies (Faisal et al., 2020a, Faisal et al., 2020b).

The World Health Organization estimates that more than 2 billion people throughout the world are prone to dengue fever. Recently, substantial rise have been witnessed in the outbreaks of the fever with major symptoms including nausea, severe headache, occasional vomiting and limbs and joints pain to name a few. The dengue virus (DENV) is transferred into the bodies of mammals (humans) through the bite of female mosquitos known as *Aedes Aegypti* (Simo et al. 2019). The lifespan of the mosquito is brief but after the larvae has the potential of spreading the virus from 4 to 10 days (Ahmed et al. 2020). Our study concludes promising potential of biosynthesized NPs as an effective and affordable approach to control the *Aedes Aegypti* and thus of the dengue fever. Both the NPs resulted in excellent larvicidal activity with Co_3_O_4_-NPs induced 67.2 ± 1.7% mortality of the larvae while MgO-NPs resulted in 49.2 ± 8.1% mortality at 400 ppm.

Co_3_O_4_-NPs and MgO-NPs were also exploited as an alternative tool for Leishmaniasis. Leshmania, is a tropical and subtropical disease mainly caused by an intracellular parasite (*Leishmania tropica*) that is transmitted to humans by the bite of a sand fly in particular Phlebotomus and Lutzomyia (Ibarra-Meneses et al. 2019). According to recent report by the World Health Organization (WHO), leishmaniasis is a serious global health issue that presents a broad spectrum of clinical manifestations with a potentially fatal outcome. The report estimates 1.5 to 2 million annual cases with 350 million people at risk of acquiring the disease (Roatt et al. 2020). Both the NPs displayed dose dependent larvicidal activity against the parasite, *Leishmania tropica*. Co_3_O_4_-NPs exhibited highest larvicidal potential with 67.2 ± 1.7% mortality while MgO-NPs resulted in 49.2 ± 8.1% mortality at the highest tested concentration (400 ppm). The varied biointeractions of the NPs may be due to their different sizes and surface chemistries. Taking into account the remarkable antileishmanial activity, our study thus concludes that Co_3_O_4_-NPs and MgO-NPs could be exploited as a possible future remedy of cutaneous leishmaniasis.

Diabetis Melitus (DM) represents a group of metabolic diseases that is mainly characterized by chronic hyperglycemia. The defect results from the reduced production of insulin or inactivity of insulin towards the body cells (Nair et al., 2018). According to the figures from International Diabetes Federation (IDF), more than 400 million individuals are affected with DM and the numbers are estimated to surge up to 600 million by 2045 (Aynalem and Zeleke, 2018). One of the key strategies to cure diabetes is to alleviate the postprandial hyperglycemia, which can be achieved by inhibiting α-amylase and α-glucosidase, the two most essential carbohydrate hydrolyzing enzymes in the digestive tract (Nair et al., 2018). At the highest tested concentration, Co_3_O_4_-NPs induced 63.19 ± 1.44% inhibition of α-amylase and MgO-NPs resulted in 54.32 ± 2.0%. Similarly, Co_3_O_4_-NPs were found to be more potent against α-glucosidase as compared to MgO-NPs with 53.27 ± 0.84% inhibition. We thus demonstrated that both the NPs can be effective alternatives to manage DM.

The antioxidant potential of Co_3_O_4_-NPs and MgO-NPs was investigated using four different assays i.e. total antioxidant capacity (TAC), total reducing power (TRP), ABTS (2,2′-azino-bis(3-ethylbenzothiazoline-6-sulfonic acid) and DPPH (2, 2-diphenyl-1-picrylhydrazyl) free radical scavenging assays (FRSA). All the bioassays affirmed excellent antioxidant property of both the NPs in particular MgO-NPs. Our study supports and augment previous studies (Kandiah et al. 2019).

The excellent biological properties of both the NPs can be attributed to two most important factors i.e. morphology and surface chemistry. For instance, size and shape are the key aspects in determining the interaction of NPs with cells (Mu et al, 2014). Studies have proved that the NPs with smaller size, exhibit enhanced antimicrobial potential due to their direct interaction with microbial membranes (Rydz et al., 2019, Kasemets et al., 2019). Moreover, green synthesis using plant extracts results in capping of phytochemicals on the NPs surface that can be an important factor in defining antioxidant and enzyme inhibition activity of the synthesized Co_3_O_4_-NPs and MgO-NPs (Hafeez et al., 2020, Abinaya et al., 2021).

Biocompatibility is one of the most important factors for the clinical viability of nanosystems. The inherent physico-chemical characteristics (such as shape, size and surface chemistry) as well as the environment in which NPs come into contact determines the degree of biocompatibility (Naahidi et al., 2013). Considering this importance, we evaluated the *in vitro* haemocompatibility of both the NPs. Both the NPs, displayed remarkable biocompatibility against the isolated human red blood cells (hRBCs) even at the highest tested dose of 400 µg/mL. Our analysis thus affirms that *Hibiscus rosa sinensis* synthesized Co_3_O_4_-NPs and MgO-NPs are highly biocompatible and can be used in multifaceted biomedical applications.

## 6 Conclusion

A facile and nonhazardous synthesis of Co_3_O_4_-NPs and MgO-NPs was reported using aqueous leaf extract of *Hibiscus rosa sinensis* as a potential reducing and stabilizing source. The FTIR analysis confirmed the successful capping of naturally occurring phytoconstituents of the plant extract. The morphological examination via SEM and TEM showed the mean size of 27.72 for MgO-NPs and 18.98 for Co_3_O_4_-NPs. The NPs were investigated for multifaceted biological applications including bactericidal activity against urinary tract infection (UTI) isolates, leishmaniasis, larvicidal, anti-diabetic antioxidant and biocompatibility studies. Our studies revealed that both the NPs were highly active against multidrug resistant UTI isolates as compared to traditional antibiotics. Both the NPs, in particular Co_3_O_4_-NPs also showed significant larvicidal and leishmanicidal activities against the *Aedes Aegypti,* the mosquitoes involve in the transmission of Dengue fever and *lesihmania tropica*, respectively. Furthermore, the NPs significantly inhibited α-amylase and α-glucosidase, the key enzymes involved in the onset of Diabetes Mellitus (DM). Last but not least, the nonhazardous and biocompatible nature, make the *Hibiscus rosa sinensis* synthesized Co_3_O_4_-NPs and MgO-NPs as green, inexpensive and potential alternatives to be exploited as for biomedical applications.

## Declaration of Competing Interest

The authors declare that they have no known competing financial interests or personal relationships that could have appeared to influence the work reported in this paper.
